# The Positive Treatment of Gonorrhœa

**Published:** 1882-11

**Authors:** 


### THE POSITIVE TREATMENT OF GONORRHŒA.

A. M. R. C. S. correspondent of the London *Lancet* gives the following account of a case of gonorrhœa observed by himself :

"Of course it is very wicked to confess that ever I had such an ailment, but truth compels me to say that, after seeing much practice at the Manchester Lock Hospital, and having duly passed my M. R. C. S. examination, and been actively engaged in practice, to my very great surprise one day I found myself (for the first time in my life) enjoying the promise of a smart attack of urethral inflammation. Now, whilst the prescribing of medicine and pocketing the reward of my industry (especially the latter), has constituted one of the chief enjoyments of my life during the last twenty years, I have to confess to an all but utter inability to swallow any of the nauseous compounds in which I daily dabble. You may fancy my exultant feelings in the prospect of having to face the stern reality of balsam, cubebs, etc. Of course I immediately consulted the authorities (I think we always do when matters so nearly come home), the result being that, all things considered, I came to the conclusion that the treatment recommended in 'Druitt's Vade Mecum' was about the most sensible and practical treatment I could find, and one deserving honest trial. Of course, too, I was a bachelor, so had every opportunity of doing every justice to the two grains to the ounce of nitrate of silver injection ; but as I sat for hours after its use nursing that weak solution, and the extreme and persistent pain it occasioned, and then shrieked out with agony when forced to micturate, I reluctantly was forced to the conclusion that Dr. Druitt never had this ailment, or if he had, had certainly written his book before trying the remedy he recommended. Afterwards when suffering from severe orchitis brought on by this injection, I 'unanimously' came to the direct decision that he knew nothing at all about it. For two whole years I held courageously to a gleety discharge, set up entirely by this insane treatment ; but eventually, to get rid of it at the cost of a persistently recurring irritability of the neck of the bladder (for which I hold this so-called 'abortive treatment' entirely responsible), sulphate and chloride of zinc are all but equally painful, whilst such astringents as tannin, etc., in arresting too suddenly the discharge (which is but the product or result of the acute inflammation), are sure to be productive of as much harm as good. Now, as against this abortive treatment of this ailment, I will tell your readers of a most positive cure of gonorrhœa, one at least which, based upon a large experience, I have never known to fail, and which possesses the advantage of being as palatable as most remedies which we are called upon to use in our every day experience of other ailments. Having, as I before asserted, a somewhat considerable practice in this direction, I get one pound of roughly ground cubebs (I grind my own, and so can rely on its freshness) ; this I put into a 200 oz. or wide mouth bottle of commerce ; to this I add 2 oz. of the iodide of potash, filling up my bottle with cold water, into the mouth of which I drop several large lumps of camphor, simply to make it keep. This I shake up two or three times a day for a few days, of course keeping it in a very cool place, afterwards pouring off the clear infusion, which I administer to my patients in regular consecutive doses. —*Detroit Clinic.*

[The "abortive treatment" increases complication, suffering and time in a large number of the cases met with in general practice].—Ed.

